# Imaging of intranasal drug delivery to the brain: updated review 2.0

**DOI:** 10.1007/s13346-026-02204-8

**Published:** 2026-08-17

**Authors:** Hannah E. Beaven, Marina R. Schinker, Thura Tun Oo, Kylie R. Smith, Mosa Alhamami, Michael C. Veronesi

**Affiliations:** 1 Department of Radiology and Imaging Sciences, Indiana University School of Medicine, Indianapolis, IN USA; 2 Department of Radiology, University of Wisconsin School of Medicine and Public Health, Madison, WI USA; 3 Institute for Quantitative Health Science and Engineering, Michigan State University, East Lansing, MI USA; 4 Departments of Radiology and Medical Physics, University of Wisconsin School of Medicine and Public Health, Madison, WI USA

**Keywords:** Intranasal drug delivery, Theranostics, Multimodality imaging, Blood-brain barrier, Focused ultrasound

## Abstract

Since the first review on imaging of intranasal drug delivery (INDD) was published in 2020, interest in the topic has increased, necessitating an update, which is the basis of the current review. INDD is a promising alternative for treating central nervous system (CNS) diseases. In vivo imaging is also important for therapy assessment, both clinically and preclinically. This review details studies that further our understanding of INDD and the fate of these therapies in the CNS using in vivo imaging. We performed a literature search using the keywords “Intranasal delivery” and “Imaging” from 2020 to 2025 and summarized these findings in the current updated review. This review is not comprehensive but provides examples of recent publications that explore the various treatments under development for nose-to-brain (N2B) therapeutic delivery through imaging. Imaging will include magnetic resonance imaging (MRI), positron emission tomography (PET), single-photon emission computed tomography (SPECT), gamma scintigraphy, and computed tomography (CT) for both humans and animals. Additional specialty topics pursued experimentally in animals include in vivo optical imaging techniques, including bioluminescence and fluorescence imaging. Finally, we discuss image-guided therapeutic techniques such as focused ultrasound and conclude with future directions. In this review, we introduce each imaging modality, describe its utilization, and outline its strengths and weaknesses, specifically in the context of N2B therapeutics.

## Introduction

### Intranasal delivery of drugs to the central nervous system

Central nervous system (CNS) diseases are the leading cause of disease burden in the world, and the global adjusted life years continue to increase at a concerning rate [1]. Despite a multitude of neuroprotective compounds showing promise in preclinical studies for the treatment of CNS disorders, most fail clinical translation. A major challenge for CNS delivery arises from the presence of the blood-brain barrier (BBB), which prevents approximately 98% of small molecules and nearly all large molecules from entering the brain [2]. The BBB insulates brain tissue from the bloodstream through capillary endothelial cells joined by tight junctions. While the BBB protects neurons from pathogens and harmful substances through both passive and active transport mechanisms, it also severely limits drug access to the CNS.

Modifying drug molecules to cross the BBB following intravenous and oral delivery is highly challenging [3]. Intranasal drug delivery (INDD) is a promising alternative to other routes of drug delivery, given the ability to bypass the BBB through transcellular and paracellular routes through nose to brain (N2B) pathways discussed in detail in the next few sections. Since INDD avoids both first-pass metabolism and the BBB, drugs can be delivered at potentially lower doses, with a more rapid onset and fewer side effects. INDD is less invasive, poses a smaller infection risk, and is more accessible to patients compared to other forms of drug delivery, leading to increased patient compliance [4]. Despite its many proposed advantages, intranasal delivery is complicated, which necessitates a detailed understanding of the nasal anatomy.

### Anatomy of nose-to-brain delivery

Biomolecules delivered N2B have been shown to reach the brain through a variety of pathways. These pathways can be differentiated by nasal region (respiratory or olfactory epithelium), transport conduit (blood vessels, nerves, or neurons), and type of transport (pericellular, transcellular, or intracellular). In general, intra-pathways involve transport within a defined structure, such as a blood vessel or neuron, whereas peri-pathways involve migration through the surrounding extracellular or perineural space.

**Fig. 1:**
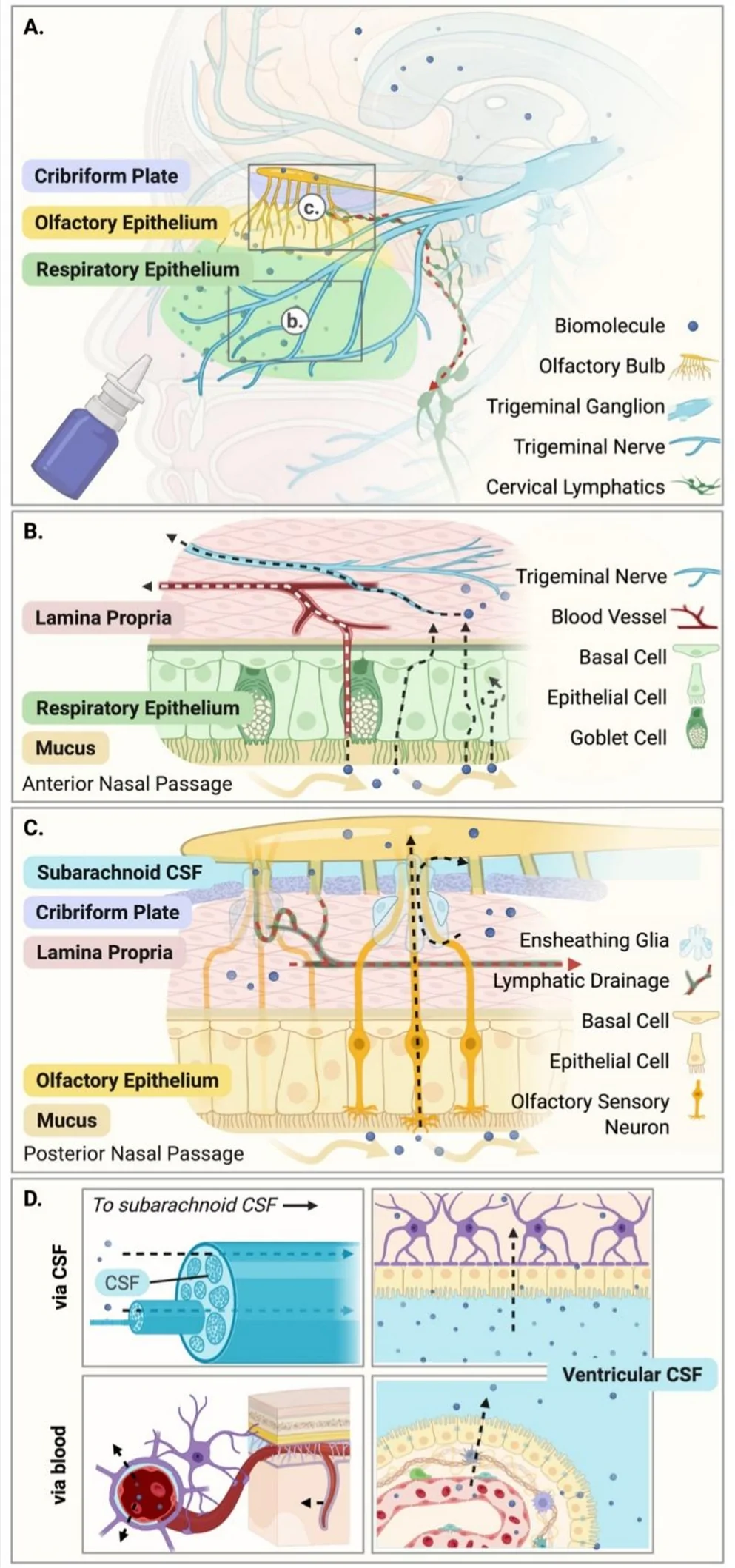
Possible fates for molecules delivered intranasally. (**a**) Overview of the N2B pathway following intranasal delivery of a molecule. Deposition and absorption may occur in the respiratory (inset b) or olfactory epithelium (inset c). **(b**) Magnified view of the anteriorly located respiratory epithelium (inset b). Molecules or drugs may cross the epithelial barrier by transcellular, pericellular, or intracellular transport to enter the lamina propria. Molecules in the lamina propria can be taken up by blood vessels and/or trigeminal nerve endings for transport to a secondary site, with potential for delivery to parenchyma. **(c)** Magnified view of the posteriorly located olfactory epithelium (inset c). Molecules endocytosed by OSNs travel intra-neuronally to directly enter the cortex where the axon synapses within the olfactory bulb (intracellular transport). Additionally, molecules may reach the lamina propria by transcellular or pericellular transport across the olfactory epithelium, subsequently reaching the intracranial compartment via bulk flow within glial ensheathments that pass through the cribriform plate (perineuronal transport). Molecules in the lamina propria may be cleared by lymphatics before transport to the brain. **(d)** Routes permitting indirect access to parenchyma via CSF (top row) or blood (bottom row) for intranasally-delivered molecules not deposited at the olfactory epithelium. Molecules absorbed by trigeminal nerve endings are transported within perineuronal spaces to trigeminal projection sites (e.g., brainstem) via fluid reportedly continuous with subarachnoid CSF (top left), enabling rapid and widespread distribution in the brain via meningeal lymphatics and the glymphatic system. Molecules in the bloodstream can access perivascular spaces (between blood vessels and the glia limitans) and utilize this meningeal/glymphatic distribution network or cross the BBB directly into the parenchyma (bottom left). Alternatively, systemically absorbed molecules may be filtered into newly produced CSF by the choroid plexus (bottom right) and subsequently reach neural tissue via transependymal flow across the ventricular border (top right). Dotted arrows indicate transport pathways. Created in BioRender. Schinker, M. (2026) https://BioRender.com/wdm4593

Following intranasal administration, a biomolecule (or drug) that lands on the anterior-inferior nasal region and traverses the mucus will reach the respiratory epithelium (Fig. 1A and B). This epithelial layer is lined by a pseudostratified ciliated epithelium interspersed with mucus-secreting goblet cells and is highly vascularized to facilitate heat and moisture exchange for inspired air, a physiological demand that necessitates a dense capillary network [5]. Biomolecules traverse the epithelial barrier to reach the vascular supply via one of several mechanisms: pericellular transport between adjacent epithelial cells, transcellular transport through the cell body from apical to basolateral membranes, or intracellular uptake into endosomal or lysosomal compartments, which may sequester molecules and slow delivery [6]. Vascular uptake allows for systemic absorption into the bloodstream and subsequent crossing of the BBB, if capable, or filtration into newly produced cerebral spinal fluid (CSF), same as required for intravenous or oral delivery (Fig. 1D) [7]. Trigeminal nerve endings are embedded within the respiratory mucosa and provide a direct route to the brainstem via intraneuronal transport (along axons) or perineural transport (through sub-epineurial structures) [8, 9]. Molecules taken up by nasal lymphatics are largely diverted to the deep cervical lymph nodes, serving as sites of immune surveillance and clearance rather than CNS delivery [10] (Fig. 1A and B).

Biomolecules that reach the posterior-superior nasal cavity will encounter the olfactory epithelium (OE), which is composed of olfactory sensory neurons (OSNs) interspersed with sustentacular and basal cells (Fig. 1A and C). OSNs extend dendrites into the nasal lumen, where inhaled air passes, and project axons that synapse in the olfactory bulb (OB) through perforations in the cribriform plate [11]. Intracellular uptake by OSNs will facilitate direct transport to the OB, making the olfactory nerve one of the most efficient intranasal-to-brain conduits [12]. Glia surrounding the OSN projection bundles passing into the intracranial compartment form a glial sheath experimentally demonstrated to be continuous with subarachnoid CSF [13]. Like with perineuronal transport, this path allows for extracellular bulk flow of solutes that may be driven by arterial pulsations inducing a “perivascular pump” effect [14] (Fig. 1C). While intracellular transport can require hours to days, extracellular perineural or perivascular transport occurs within minutes and is predicted to dominate many INDD applications [15]. Recent evidence indicates that intracellular and extracellular mechanisms can occur simultaneously, with some molecules utilizing multiple routes, further complicating pharmacokinetic predictions [16].

Molecules that reach the subarachnoid CSF become subject to widespread distribution and clearance throughout the brain via meningeal and glymphatic networks. Astrocyte-bound perivascular spaces form part of the glymphatic network, where subarachnoid CSF penetrates the parenchyma along periarterial channels, mixes with interstitial fluid to distribute solutes and clear waste, and exits along perivenous routes, thus facilitating exchange between perivascular CSF and interstitial fluid [17]. Once in the interstitium, biomolecules may exert their effect on neurons and/or glia. N2B agents have been found to have unique access to this distribution network, particularly following transfer along olfactory and trigeminal nerves, where fluid continuous with subarachnoid CSF fills perineuronal and sub-epineurial spaces [18] (Fig. 1D). For this mode of access, brain penetration depends on molecular size and polarity (small, lipophilic molecules diffuse more readily), CSF flow dynamics and pressure gradients, as well as astrocytic aquaporin-4 (AQP4) activity and arousal state, which modulate glymphatic function [19]. While this pathway primarily clears CSF and macromolecules from the brain [10], it also creates a potential barrier to drug entry affected by local pressure gradients and molecular properties, underscoring the dynamic interplay between nasal lymphatics, meningeal drainage, and perineuronal transport in shaping intranasal pharmacokinetics [19, 20].

### Approved and clinically promising drugs for INDD

As of October 2025, the FDA Orange Book of approved drug products lists 121 currently available therapies with “nasal” as the route of delivery [21]. Forty unique active ingredient combinations are represented across the different products, including thirty-two prescription-only drugs and eight over-the-counter formulations. A summary of these drugs is provided in the following paragraphs, with indications obtained from the FDA Label database unless otherwise indicated [22].

The most common indication for current FDA-approved nasal therapies is allergic rhinitis, which applies to 13 locally-acting drugs, including budesonide, olopatadine hydrochloride, cromolyn sodium, and fluticasone furoate, to name a few examples. Related indications include oxymetazoline hydrochloride-tetracaine hydrochloride combination therapy for nasal decongestion, mometasone furoate for chronic rhinosinusitis, and ipratropium bromide for cold- and allergy-related rhinorrhea. Yet another drug targeting the nasal cavity itself is cocaine hydrochloride, which functions as a local anesthetic for intranasal procedures.

Other intranasally delivered drugs are absorbed systemically and have a range of indications, including migraine (dihydroergotamine mesylate, sumatriptan, zavegepant, and zolmitriptan), pain relief (butorphanol tartrate, ketorolac tromethamine), opioid overdose reversal (nalmefene hydrochloride, naloxone hydrochloride), and epilepsy (diazepam, midazolam). Intranasal calcitonin salmon is used for postmenopausal osteoporosis; cyanocobalamin for vitamin B12 deficiency; desmopressin acetate for central diabetes insipidus; esketamine hydrochloride for treatment-resistant depression; glucagon for severe hypoglycemia in diabetic patients; metoclopramide hydrochloride for recurrent diabetic gastroparesis; nafarelin acetate for endometriosis in adult women and central precocious puberty in children; nicotine for smoking cessation therapy; testosterone for primary hypogonadism; and varenicline for dry eye disease. Other therapies newly approved in 2025 include intranasal epinephrine for anaphylaxis and intranasal bumetanide for edema in the setting of congestive heart failure, hepatic, or renal disease [23].

A plethora of other drugs have been studied preclinically but have not yet advanced down the drug development pipeline, including some that may be able to undergo direct nose-to-brain delivery. Notable candidates for this route of delivery include chemotherapeutic agents for glioblastoma, oxytocin for social behavior, orexin-A for narcolepsy, leptin for obesity and sleep disorders, insulin for addressing cognition in Alzheimer’s disease (AD) and mild cognitive impairment (MCI), and siRNAs and stem cells for treating neurodegenerative diseases [24]. Research in both formulation and delivery is needed for these potential drugs to be fully realized as safe, efficacious, and potent therapies.

## Imaging and optimization of INDD

Successful INDD depends on many factors, including delivery technique, drug formulation, and the physiological state of the nasal environment and tissues. In both animal models and human studies, imaging modalities have become instrumental for understanding how each of these variables can be modified to alter INDD outcomes. In one example focused on device performance, imaging was used to demonstrate superior targeting of the posterior nasal cavity in humans when participants used a breath-powered nasal delivery device compared to a hand-actuated liquid sprayer [25]. In a different example focused on formulation, Ahmad et al. utilized imaging to investigate whether chitosan, a known absorption enhancer and tight junction opener [24], could improve nanoemulsion retention in the nasal cavity [26]. In yet another example leveraging physiology, Ye et al. examined whether INDD could be augmented with a theranostic ultrasound technique to improve CNS localization by modulating blood vessel oscillation [27]. These examples illustrate the essential role of imaging for optimizing delivery methods that minimize toxicity and maximize therapeutic efficacy.

**Fig. 2:**
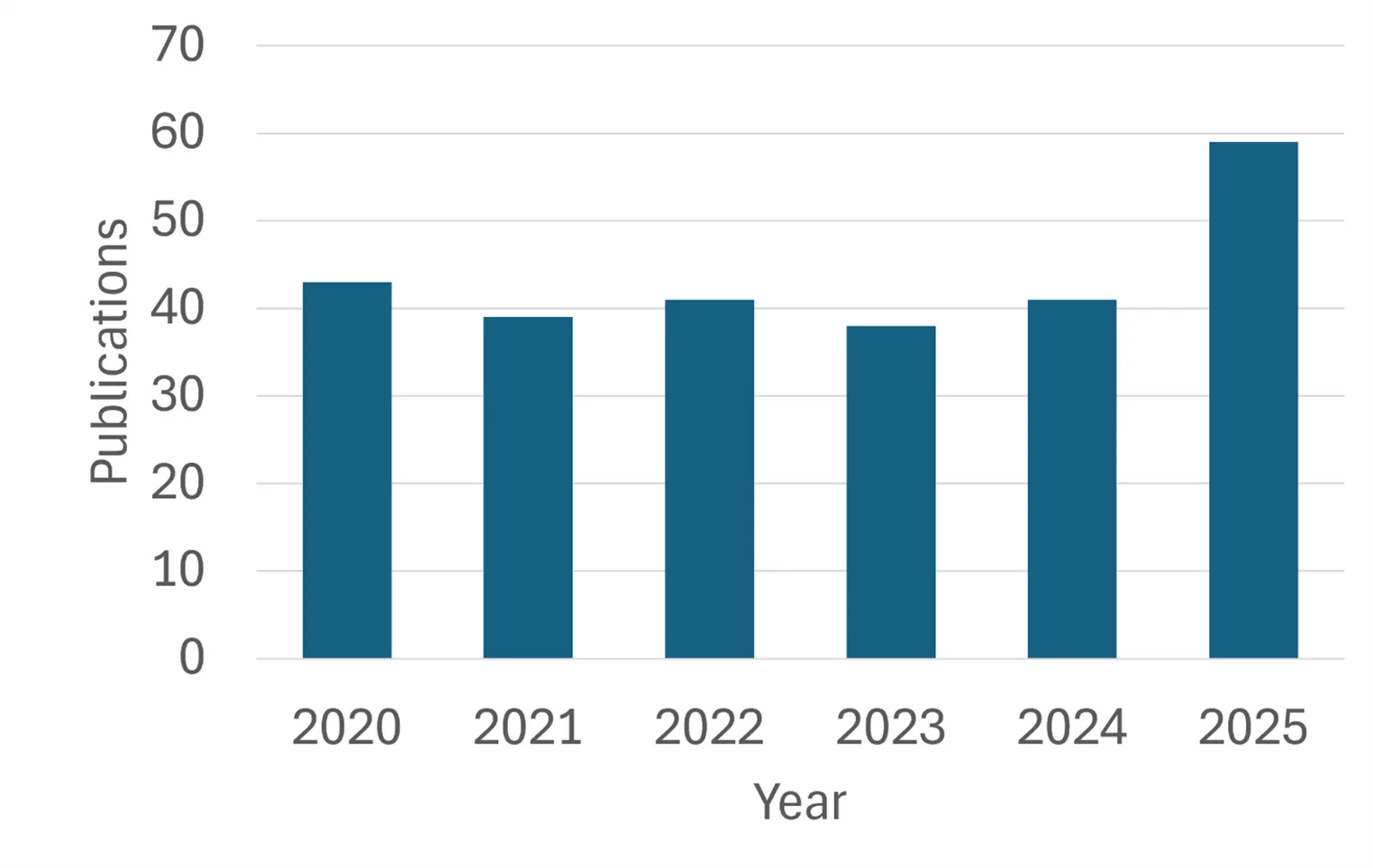
INDD publication trends from 2020–2025. Bars correspond to the total publication count each year based on a Pubmed search (pubmed.gov) with the search query “intranasal delivery imaging”

The utility and versatility of the imaging toolkit as a means of studying INDD is reflected in the steady increase in publications with the keywords “intranasal delivery” and “imaging” over the past two decades, with over 200 papers published in this area since 2020 (Fig. 2). The first review on imaging of INDD was published in 2020 [28]. While the expanding body of literature has been thoughtfully synthesized in recent systematic registers that catalog decades of historical developments and specific radioisotope chemistry [29], there remains a distinct need for a framework focused on operational translation. Rather than compiling a retrospective registry of tracer modalities, this updated review isolates the advancements occurring between 2020 and 2025 to evaluate how delivery devices, advanced formulations, and physiological barriers are actively manipulated to maximize central nervous system target engagement. The emphasis here centers on the transition from proof-of-concept animal models to active, image-guided therapies and first-in-human clinical evaluations.

In this review of studies published from 2020 to mid 2025, we introduce each imaging modality, describe its utilization, and outline its strengths and weaknesses in the context of studying N2B delivery. Key illustrative experiments are again highlighted which utilize magnetic imaging resonance (MRI), positron emission tomography (PET), single-photon emission computed tomography (SPECT), gamma scintigraphy, and computed tomography (CT) to perform in vivo study of INDD in both humans and animals. Experimental specialty techniques in optical imaging are also covered, including bioluminescence and fluorescence imaging. Finally, we touch on relevant emerging image-guided drug delivery approaches such as focused ultrasound, and conclude with a translation-focused discussion of future directions.

**Table 1:** Intranasal imaging with MRI

Author	Year	Technique	Species	Condition	Goal	Key Finding
Wagenaar et al. [30]	2025	MR Volumetry	Humans	Stroke	Assess IN delivery of MSCs after stroke on brain tissue loss, adverse events, and neurological outcomes in neonates.	Damage to the corticospinal tract was reduced in IN MSC subjects after 3 mos. Motor performance was better than untreated group at 2 yrs.
O’ Brien et al. [31]	2025	fMRI	Humans	Neuro-Psychiatric	Assess if IN oxytocin modulates brain activity during emotional processing in children with treatment resistant conduct problems.	Activation in the posterior cingulate and precuneus to happy faces suggested IN oxytocin may promote a neurotypical profile in conduct problems.
Zhang et al. [32]	2025	Anatomic	Mouse	Stroke	Deliver intranasal nanosuspension to improve neurological outcomes in mice following MCA infarction.	A fingolimod-based IN nanosuspension significantly lowered infarct volume in treated versus untreated mice.
Ye et al. [33]	2024	MR CEST	Mouse	AD	Investigate whether IN oxytocin normalizes intracranial lymphatic drainage in aged AD mice.	IN oxytocin administration was associated with improved glymphatic and meningeal lymphatic drainage, as well as improvements in cognitive and memory deficits.
Gao et al. [34]	2024	Anatomic	Rats	Nasal Toxicity	Evaluate the toxicity of ZnO NPs on the OE of rats.	MRI showed structural alterations and inflammation in the OE and OB following exposure to 20-nm ZnO NPs, while OB toxicity from ZnOs was size-dependent.
Law et al. [35]	2023	MR CEST	Mouse	Fate Mapping	Leverage CEST to study mucus-penetrating liposomes loaded with iohexol following intranasal delivery.	Higher CEST contrast between the OBs and the OB external plexiform layer, relative to the deeper granule cell layer.
Korann et al. [36]	2022	fMRI	Humans	Neuro-Psychiatric	Resting state functional MR imaging study looking at connectivity changes following IN oxytocin dosing in patients with schizophrenia.	Connections between the left caudate and supplementary motor area, left precentral gyrus, and left frontal inferior triangular gyrus were significant.
Wingrove et al. [37]	2022	fMRI	Humans	Appetite/obesity	Study IN insulin effects on anticipatory and consummatory responses to sweet stimuli in normal vs. overweight subjects.	IN insulin produced differences in normal vs. overweight subjects in certain brain regions.
Kellar et al. [38]	2021	Anatomic	Humans	MCI/AD	Evaluate whether IN insulin can slow cognitive decline in patients with AD and MCI.	IN insulin reduced pathologic increases in white matter hyperintensity volume within frontal lobe regions and deep white matter.
Craft et al. [39]	2020	MR Volumetry	Humans	MCI and AD	Evaluate IN insulin as a treatment for MCI and AD using two nasal delivery devices including brain volumes.	Small but significant reduction in entorhinal cortex volumes was observed in IN insulin treated group.

**Table 2:** Intranasal imaging with PET

Author	Year	Technique	Species	Condition	Goal	Key Finding
Huang et al. [40]	2025	^18^F-GE-180 TSPO PET	Rats	Acute spinal cord injury	Assess the biodistribution of IN delivery of BDNF-sEVs. Determine if sEVs reduce neuroinflammation/improve neural repair.	IN delivered sEVs accumulated in the injured spinal cord, with a significant reduction in TSPO PET (marker of microglial activity).
Solingapuram et al. [41]	2025	^68^Ga-NOTA-Insulin PET	Humans	Fate Mapping in MCI	Evaluate safety and kinetics of IN delivery of a novel PET tracer the brains of patients with MCI versus healthy aging controls.	Patients with MCI had lower insulin activity in all brain regions compared to control. The concentration decreased after the 1st timepoint.
Morin et al. [42]	2025	^18^F-FLT PET	Rats	Fate Mapping	Validate that the Olfactory Drug Delivery (ODD) method is capable of delivering a radiotracer to the olfactory cleft and into the brain in rodents.	ODD provided superior frontal brain delivery of the poorly BBB-permeable tracer [¹⁸F]FLT at early time points compared to traditional IN and IV administration.
Gotoh et al. [43]	2025	^64^Cu-NOTA-exosome PET	Rats	Subarachnoid Hemorrhage	Investigate biodistribution and therapeutic potential of exosomes released from mesenchymal stem cells.	PET imaging confirmed ^64^Cu exosome translocation to the cerebrum via olfactory and trigeminal pathways, with peak accumulation in the olfactory nerve (~ 1 × 10^5^ particles) and the damaged cerebral hemisphere.
Singhal et al. [44]	2025	^18^F-PBR06 and 18F-FlorbetapirPET	Human	AD	Evaluate impact of nasal-foralumab treatment on microglial activation as assessed by 18 F-PBR06-PET in case study of an elderly man with AD.	3 months of nasal-foralumab treatment reduced microglial activation based on ^18^F-PBR06-PET and reduced the amyloid burden based on amyloid PET.
Smith et al. [45]	2024	^18^F-Insulin PET	Primates	Fate Mapping	Apply PET imaging to characterize the distribution of [F-18]FB-insulin in the nasal cavity and brain of primates following 3 modes of IN delivery.	[F-18]FB-insulin crossed the cribriform plate and was detected in all brain regions after IN delivery, esp. elevated levels in involved in emotional and memory processing.
Solingapuram et al. [46]	2024	^68^Ga-Insulin PET	Primates	Fate Mapping	Validate nose-to-brain transfer of Ga-68-labeled-insulin and characterize whole-body dosimetry in primates via PET.	PET demonstrated an acceptable dosimetry profile and brain penetration of the Ga-68-labeled-insulin.
Fukakusa et al. [47]	2024	^11^C-Raclopride PET	Rats	Fate Mapping	Investigate the permeation enhancer lauroylcholine chloride (LCC) for improved IN delivery of domperidone to the brain imaged with dopamine D2 receptor (D2R) PET.	Administering LCC with domperidone increased its delivery to the brain by 2.4-fold as measured with D2R targeting PET.
Yuki et al. [48]	2023	PET	Mice and Rhesus Macaques	Fate Mapping	Assess the biodistribution of a nasally administered nanogel-based vaccine delivery system in vivo to confirm its safety.	PET imaging showed no accumulation of the nanogel in the brain or OBs of mice or non-human primates.
Veronesi et al. [4]	2021	PET	Rats	Fate Mapping	Develop a PET imaging protocol to evaluate brain distribution of 89-Zr-labeled polymeric micellar NPs (LPNPs) following aerosolized IN and IV delivery.	Aerosolized IN delivery yielded higher concentrations of 89-Zr-LPNPs in the brain at 1 and 2 h compared to IV delivery.

**Table 3:** Intranasal imaging with SPECT

Author	Year	Technique	Species	Condition	Goal	Key Finding
Morin et al. [49]	2024	SPECT	Humans	Fate Mapping	Validate upper nasal cavity targeting by novel INDD device and demonstrate functional CNS changes via fMRI that result from treatment using the novel device.	Predominant deposition at the olfactory cleft was observed in 7/8 subjects following delivery of a Tc99m-labeled radiotracer via a novel device utilizing laminar flow ejection (LFE) as the INDD method.
Simón et al. [50]	2023	SPECT	Rats	Fate mapping	Characterize the release of radiolabeled risperidone from a nasal implant as a proof-of-concept for sustained INDD.	SPECT/CT tracked the steady release rate of radiolabeled risperidone (I-125) from nasal rods implanted into the rat nasal cavity over 4 weeks. Release rate (as related to the reduction in SPECT signal) was slow and steady for all tested compounds but was slower for the free I-125 than the radiolabeled drug.

**Table 4:** Intranasal imaging with fluorescence imaging

Author	Year	Technique	Species	Condition	Goal	Key Finding
Brar et al. [51]	2025	FLI	Mouse	Glioblastoma	Apply in vivo fluorescence imaging to study an IN micelle therapy for glioblastoma.	Micelles localized to the tumor-bearing region of the brain with reduced accumulation in body tissues compared to an IN drug solution.
Shen et al. [52]	2025	FLI	Mouse	Obesity	Apply FLI and BLI to study the IN delivery of engineered, hormone-secreting commensal bacteria as a treatment for obesity.	FITC-tagged bacteria gradually accumulated in the OB while secreted hormones appeared intracellularly throughout all layers of the OE.
Bolon et al. [53]	2025	FLI	Mouse	None	Evaluate the N2B delivery efficacy of plain and poloxamer-coated nanoparticle (NPs).	P188-coated NPs exhibited the highest in vivo fluorescent signal intensity, although this difference was not statistically significant.
Zhao et al. [54]	2023	FLI	Mouse	PD	Determine whether IN delivery of a quercetin-based nanomedicine could treat PD symptoms in mice.	Ex vivo confocal microscopy confirmed delivery to the brain, and FLI showed peak drug signal at 1 h.
Tolomeo et al. [55]	2023	FLI	Mouse	None	Evaluate the kinetics and biodistribution of MSC-EVs following IN, IT, and IV delivery.	In vivo signal after IN delivery was visible at 3 and 24 h, with lower off-target accumulation in the lungs and liver relative to IT and IV delivery.
Han et al. [56]	2023	FLI + SPECT-CT	Mouse	Glioma	Elucidate the transport of fluorescently tagged gold nanorods in a glioma mouse model.	Frontal brain region uptake was visualized with off-target accumulation in the stomach and intestines.
Cheng et al. [57]	2023	FLI + MRI	Mouse	AD	Evaluate the ability of oxytocin-loaded, macrophage-biomimetic liposomes to ameliorate Alzheimer’s symptoms in mice.	Brain localization on FLI was corroborated by IF staining; MRI showed improved hippocampal volumes with parallel memory improvements.
Perets et al. [58]	2019	FLI + CT	Mouse	AD, PD, Stroke, and Autism	Investigate the IN administration of mesenchymal stem cell -derived exosomes (MSC-exos) for the treatment of CNS disorders.	Gold nanoparticle-labeled MSC-exos localized to pathological tissue regions in mouse models of AD, PD, stroke, and autism.

**Table 5:** Intranasal imaging with bioluminescence imaging

Author	Year	Technique	Species	Condition	Goal	Key Finding
Villa et al. [59]	2024	BLI + FLI + MRI	Mouse	Glioma	Investigate the ability of canine glioma-derived extracellular vesicles (CG-EVs) to target glioma tumors in mice.	CG-EVs preferentially accumulated in both peripheral subcutaneous and brain gliomas.
Wang et al. [60]	2020	BLI + PET/CT	Mouse + Rat	PD	Determine the detection limits of BLI and PET/CT when delivering a human neural stem cell (hNSC)-based therapy for PD.	Using BLI, a concentration of 1 × 10^6^ hNSCs was required for detection at 20 min in rats and 1 × 10^5^ cells in mice. Using PET/CT, a dose of 2 × 10^6^ ^89^Zr-hNSC was required for detection at 4 h post-IN delivery.

**Table 6:** Focused ultrasound (FUS) and image-guided drug delivery

Author	Year	Technique	Species	Condition	Goal	Key Finding
Lin et al. [61]	2025	FUS	Mouse	None	Test the ability of low-intensity, transcranial planar ultrasound to improve the intranasal uptake of fluorescently-tagged dextran.	Higher fluorescence levels were observed in the trigeminal nerve and OBs when using US compared to IN delivery alone.
Semyachkina-Glushkovskaya et al. [62]	2022	Photo-stimulation	Mouse	Glioblastoma	Investigate the ability of pulsed laser light to optimize the intranasal treatment of glioblastoma with ganglioside GM1-containing liposomes.	Pulsed laser light treatment improved the lymphatic spreading of liposomes in glioblastoma-afflicted mice.
Pardo et al. [63]	2021	External magnetic field	Mouse	None	Improve N2B delivery of magnetoelectric nanoparticles (MEN) via the application of an external magnetic field (EMF).	Both 30 and 60 nm MENs were observed in upper brain regions when an EMF was applied for 30 min following INDD, whereas uptake of 60 nm MENs was minimal when EMF was not applied.
Pandey et al. [64]	2020	External magnetic field	Rats	Glioblastoma	Evaluate a mitochondria-targeted, multimodal glioblastoma therapy consisting of pH-responsive, coated FePt core shell NPs delivered intranasally.	FITC-labeled NPs were delivered to the OE and brain tissues, although with significant off-target accumulation in the stomach and intestines.
Seino et al. [65]	2024	External magnetic field	Mouse	AD	Evaluate the N2B delivery of surface-modified superparamagnetic iron oxide NPs for magnetic particle imaging.	NPs were observed to accumulate in senile plaques at 6- and 48-hours following delivery. Between 0.26–0.62% of the delivered dose was detected in the brain.

## Magnetic resonance imaging (MRI)

Magnetic resonance imaging (MRI) is a non-ionizing technique based on nuclear magnetic resonance that uses a powerful magnet and radio waves to create non-invasive, detailed anatomical images of the body. Quantitative and functional MR techniques are a cornerstone of diagnosis and treatment assessment. Clinical scanners typically operate at 1.5–3 tesla, newer ultra-high-field systems reach 7 tesla (now FDA-approved for human use), and small-animal systems can approach ~ 21 tesla (although typically range from 4.7 to 9.4 tesla). During a scan, the magnet aligns hydrogen nuclei in tissues; short radio-wave pulses disturb that alignment, and the tiny signals emitted as the hydrogen nuclei realign to the magnetic field are captured by receiver coils. Additional coils subtly vary the magnetic field to encode position and select slices. Together, these steps produce high-resolution, multiplanar images with excellent soft tissue contrast without exposing patients to ionizing radiation [66]. Image contrast in MRI arises primarily from intrinsic tissue properties: proton density, T1 (longitudinal) relaxation times, and T2 (transverse) relaxation times (including T2*). By selecting appropriate pulse sequences (and timing), MR images can be weighted to emphasize T1, T2/T2*, or proton density differences in biological tissues, which improves visualization of more subtle anatomical and pathological details. Higher magnetic field strengths generally improve signal-to-noise ratio (SNR) and spatial resolution, enabling high-detail imaging of small anatomical structures [66].

A summary of MRI-based INDD experiments is provided in Table 1. In humans, MRI is used in different ways as a useful tool to study the effects of INDD on a range of neurological diseases and conditions, including MCI and AD [38, 39] appetite and obesity [37], and neuropsychiatric conditions [31, 36]. For example, Kellar et al. used MRI to demonstrate a reduced white matter hyperintensity volume (WMHV) increase in MCI and AD patients after 12 months of twice daily INDD with 20 IU insulin, as compared with placebo. Cognitive testing revealed that increased WMHV was associated with worse memory composite scores, suggesting that INDD with insulin may be a viable therapy for slowing cognitive decline in AD and MCI patients [38]. In another interesting clinical trial, the feasibility, safety, and efficacy of intranasally delivered insulin were evaluated using two different FDA-approved nasal delivery devices. Along with improvement in cognitive performance and activities of daily living scores, a small but significant reduction in entorhinal cortex volumes was observed by MRI in subjects treated with the Kurve ViaNase device, a switch-actuated, metered-dose nasal sprayer utilizing liquid hydrofluoroalkane propellant, compared with placebo [39]. The impact of intranasal mesenchymal stromal cells (MSCs) was studied as a potential treatment for neonatal arterial ischemic stroke using MRI. Participants in the PASSIoN Trial (Perinatal Arterial Stroke Treated with Stromal Cells Intranasally) showed no adverse events, greater preservation of brain tissue, and improved motor outcomes compared to registry controls that didn’t receive IN MSC treatment [30].

Preclinically, Zhang et al. used MRI and behavioral testing to study the impact of a nanosuspension of fingolimod and curcumin on neurological outcomes in mice following cerebral infarction. After optimizing delivery to the posterior nasal cavity using 3D printed nasal cast models, mice were inoculated with the nanosuspension at varying time points after a surgically induced middle cerebral artery (MCA) infarct. Subsequent MRI imaging demonstrated lower infarct volume in treated mice at 48 h and superior performance on behavioral tests at 2- and 3- days post-stroke compared with untreated controls [32]. The authors concluded that while many translational hurdles remain, including differences in nasal anatomy and applicability to other types of strokes, INDD with nanosuspension technology could serve as a promising therapeutic strategy to improve neurological recovery following cerebral vascular occlusion.

**Fig. 3:**
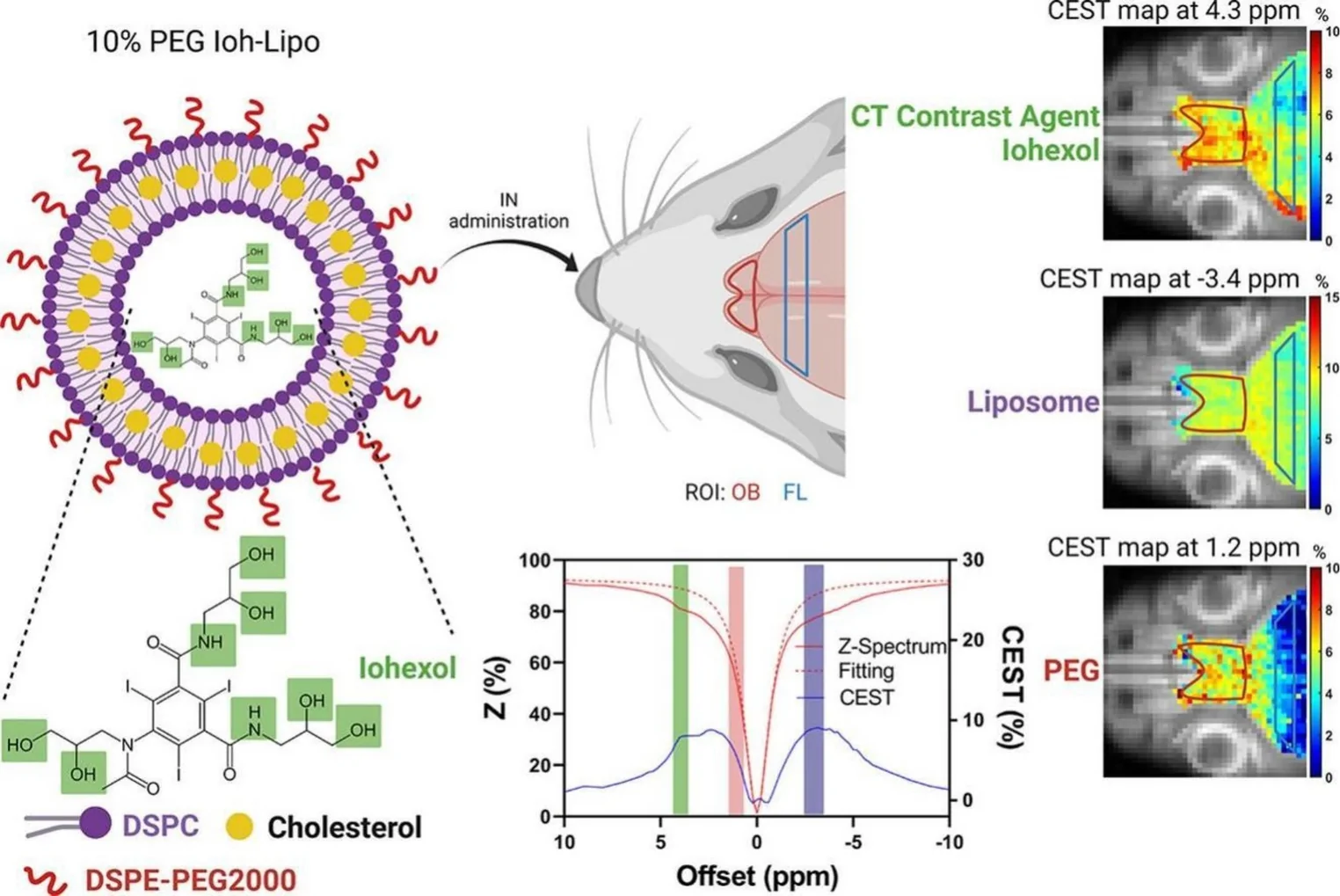
MRI study of INDD using multiple CEST contrast imaging. Exogenous CEST contrast agent iohexol was loaded into mucus-penetrating liposomes composed of 10% polyethylene glycol. Distributions in the brain were then measured using CEST maps at 4.3 ppm, − 3.4 ppm, and 1.2 ppm. Reproduced from Law et al., *Journal of Controlled Release*; published by Elsevier, 2023

Law et al. leveraged MR-based chemical exchange saturation transfer (CEST) to study the INDD of mucus-penetrating PEG liposomes loaded with iohexol, a contrast agent with CEST-detectable protons. In CEST, a long saturation pulse is applied at the resonant frequency of the protons of the solute of interest, such as iohexol. This pulse equalizes the number of spins aligned with the magnetic field and the number of spins aligned against it, resulting in a loss of net magnetization and signal termed “saturation” [67]. If the solute protons have a sufficiently fast exchange rate and long saturation time, the exchange of saturated solute protons with water protons produces a detectable reduction in water signal, which can be used to calculate the concentration of solute [68]. When CEST MR imaging was performed on mice after INDD of a solution containing liposomes, CEST contrast allowed detection of maximally increased amide protons (at 4.3 ppm) and aliphatic protein content (at -3.4 ppm) in the OB at 30 min after delivery (Fig. 3). A more granular spatial analysis of signal distribution indicated significantly higher CEST contrast in the combined middle region between the left and right OBs and the OB external plexiform layer, relative to the deeper granule cell layer [35]. This finding is notable because the external plexiform layer is implicated in N2B delivery via olfactory nerve pathways, whereas the CSF supply to the middle region between OBs offers a clue as to how intranasally delivered compounds are more broadly distributed throughout the brain.

MR CEST, along with arterial spin labeling perfusion, was used to study the neuroprotective mechanism of oxytocin in a mouse model of AD following intranasal delivery [33]. Oxytocin administration modified and improved the dysfunctional glymphatic and meningeal lymphatic systems in severely impaired middle to old-aged APP/PS1 mice to facilitate improved cognition. Additionally, pathologic amyloid-beta peptide found in the AD mice was observed to drain more efficiently from the brain parenchyma into the CSF and deep cervical lymph nodes in the neck. It remains to be seen whether the success of anti-amyloid antibody therapies can be maintained if the problem of dysfunctional lymphatics persists in AD patients.

While not a direct measure of the nose-to-brain route, functional MRI (fMRI) detects brain tissue oxygen utilization to yield important information regarding the impact of various diseases and therapies on the different subregions of the brain following intranasal delivery. fMRI indirectly measures neural activity through the blood-oxygenation-level-dependent (BOLD) signal, which reflects neurovascular coupling between neuronal activation and local hemodynamics. Increased cerebral blood flow and oxygenation reduce deoxyhemoglobin, lengthen T2* relaxation, and enhance the MR signal in T2*-weighted images, producing spatially localized activation maps with a delayed temporal profile [69, 70]. An important advantage of this MRI technique is that functional observations can be made in a non-invasive manner without the need for intravenous (IV) contrast, which can be more feasibly used in human clinical and translational studies. fMRI was used to study oxytocin modulation of emotional processing in children with conduct problems [31] and in patients with schizophrenia [36] following intranasal delivery. Appetite and obesity studies are also candidate diseases that can be studied with fMRI following intranasal delivery. For example, the impact of anticipatory and consummatory effects of intranasal insulin on brain responses differed between normal and overweight subjects [37]. The usual limitations of fMRI that apply more broadly to the study of diseases and their candidate therapies include low temporal and spatial resolution, especially for smaller structures such as the OBs.

## PET

Positron emission tomography (PET) images the in vivo distribution of positron-emitting radiotracers (e.g., F-18) noninvasively by detecting pairs of 511-keV photons produced when each positron annihilates with an electron; near-simultaneous detection localizes the event along a “line of response.” Modern scanners use fast scintillators coupled to silicon photomultipliers, enabling high detection efficiency and precise timing. Incorporating time-of-flight information improves image signal-to-noise and lesion detectability [71]. Quantitative image reconstruction applies corrections for attenuation, scatter, randoms, and dead time. PET, particularly when integrated with CT or MRI, has proven to be a valuable tool for studying INDD. PET has high sensitivity, which allows for quantification of radiolabeled therapeutics in vivo, while integration with CT or MRI helps overcome lower relative anatomical resolution encountered on PET, which is essential for visualizing intricate structures like the olfactory neuroepithelium and brain subregions.

**Fig. 4:**
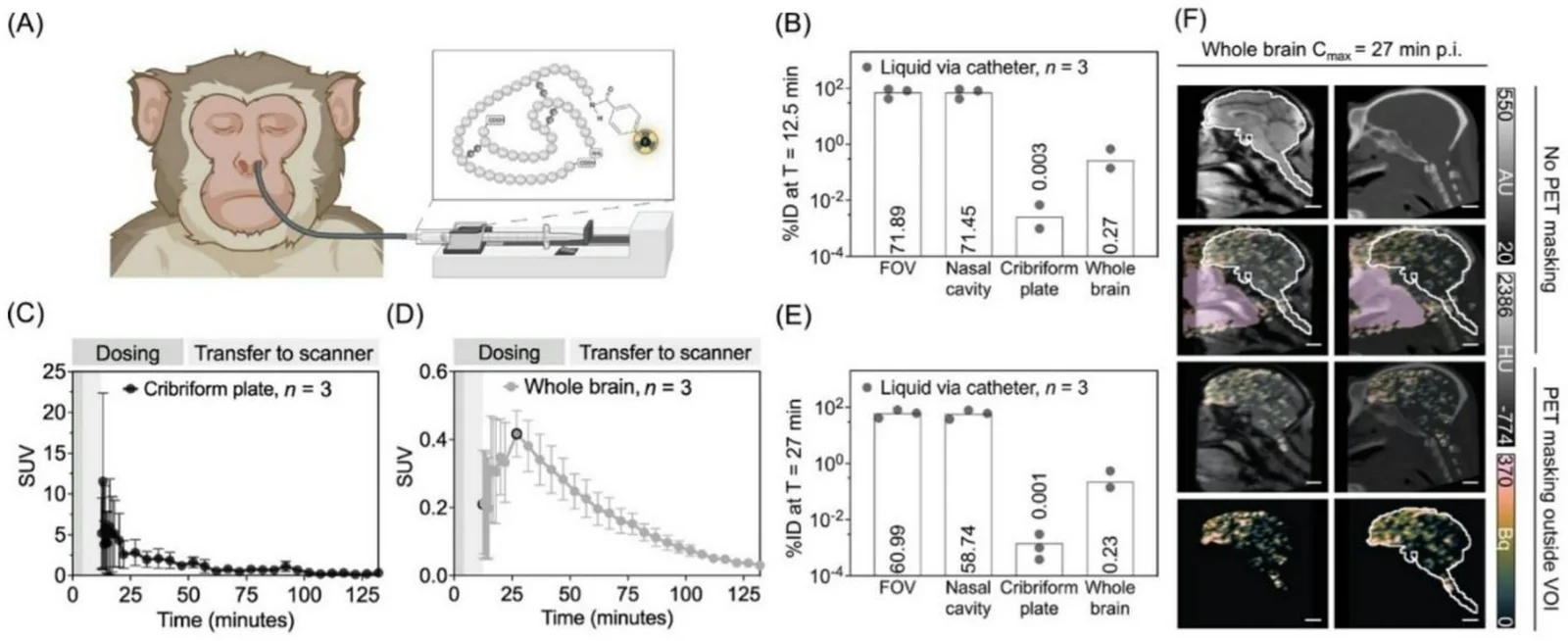
PET-CT and PET-MRI imaging of intranasal insulin. **(a)** Schematic representing intranasal delivery of [^18^F] FB-insulin via catheter. **(b)** Radiotracer levels (FOV = field of view) in the nasal cavity 12.5 min after liquid instillation. **(c)** Transfer of [^18^F] FB-insulin across the cribriform plate peaked at the earliest PET time points. **(d)** [^18^F] FB-insulin levels in the brain rose to maximum standard uptake value (SUV) by 27 min. **(e)** [^18^F] FB-insulin levels in volumes of interest at T = 27 min. **(f)** Sagittal views of co-registered CT (right column), MR (left column), and PET (both columns) at 27 min post-delivery. Adapted with permission from Smith et al., *Alzheimer’s & Dementia*; published by Wiley, 2024

At the preclinical level, exciting research is rapidly increasing in the PET realm for furthering our understanding of INDD. N2B delivery kinetics have been characterized via PET in efforts to evaluate N2B insulin as a treatment for cognitive impairment in AD. For instance, Smith and colleagues used PET following INDD of ^18^F-labeled insulin ([¹⁸F]FB-insulin) in primates to characterize its transfer kinetics and identify specific regions of localization in the brain (Fig. 4). This use of PET also allowed the group to refine and test a range of intranasal delivery strategies [45]. Alongside this effort, Solingapuram Sai et al. used PET to validate transfer and calculate dosimetry of N2B ^68^Ga-labeled insulin ([⁶⁸Ga]Ga-NOTA-insulin) in primates. The authors found that the ^68^Ga-labeled insulin delivered via a multidose nasal spray pump (Aptar Cartridge Pump System) was safe and effective for observing insulin brain transfer in vervet monkeys, corroborated limbic uptake patterns observed for [¹⁸F]FB-insulin, and provided a reliable delivery system for use in future imaging studies [46].

In the most recent clinical advances in human subjects, INDD has moved from proof-of-concept to demonstrable therapeutic efficacy in the CNS using PET imaging. In a first-in-human study, Solingapuram Sai et al. confirmed the uptake of intranasally delivered insulin to specific brain regions, including the olfactory cortex, mesial temporal structures, basal ganglia, thalamus, and anterior cingulum using the [^68^Ga]Ga-NOTA-insulin PET radiotracer. The authors demonstrated direct N2B delivery by showing that the radiotracer rapidly reached the brain while bypassing systemic circulation, as evidenced by stable blood insulin and glucose levels [41]. In a different clinical validation study, a fully human monoclonal antibody (foralumab) administered intranasally for three months resulted in decreased amyloid burden based on ^18^F-Florebetaben PET imaging. Inflammation was also decreased following PET imaging with ^18^F-Fluoroacetyl-N-(2,5-dimethoxybenzyl)-2-phenoxyaniline (^18^F-PBR06-PET), which is a second-generation 18-kDa-translocator protein (TSPO) ligand targeting microglia [44]. This study demonstrates how PET imaging can be leveraged to validate INDD treatment efficacy without imaging the drug itself.

**Fig. 5:**
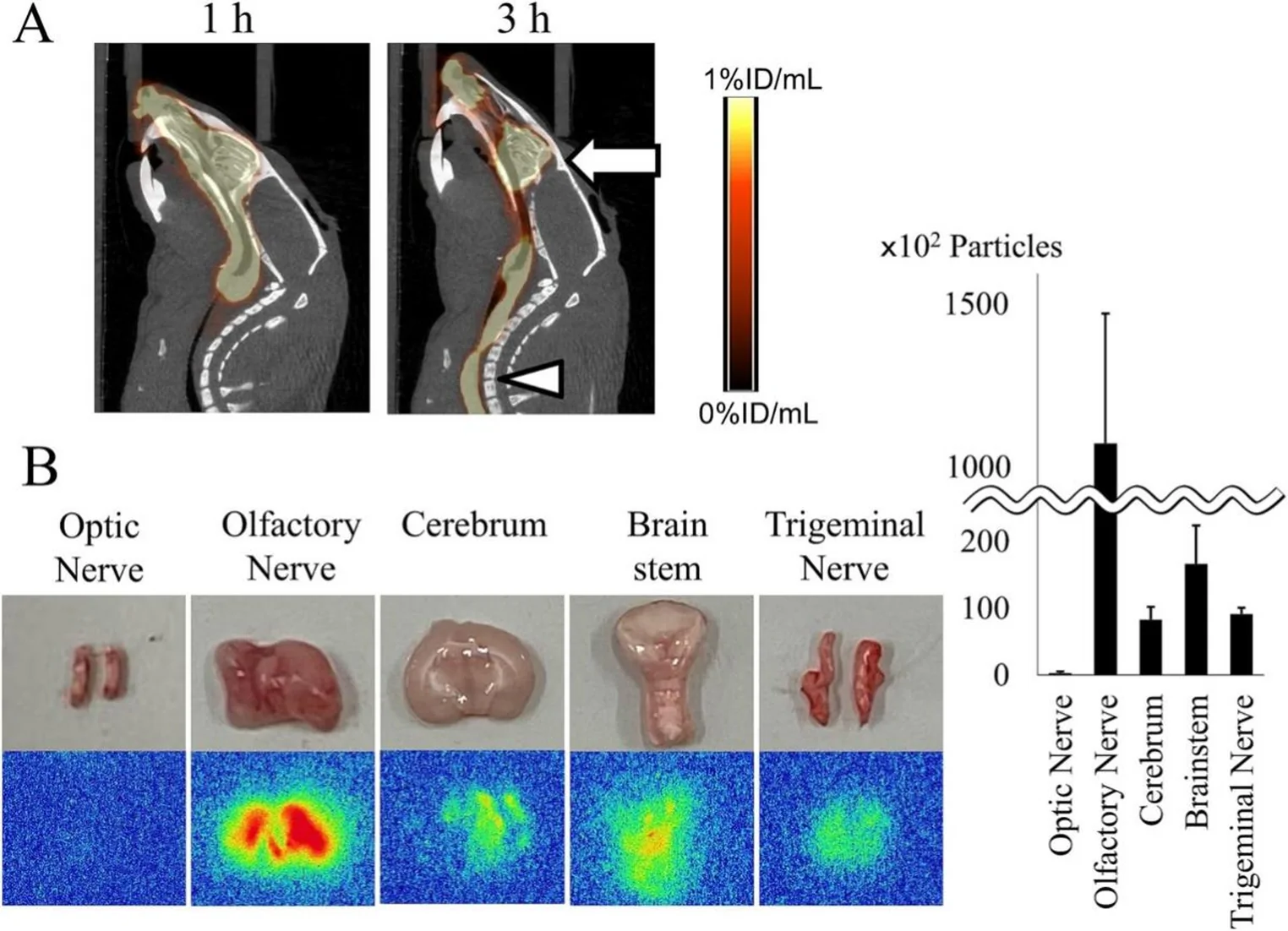
Biodistribution of intranasally-delivered exosomes using PET. **(a)** PET/CT revealed ^64^Cu-labeled exosomes were mainly observed in the nasal cavity one hour after administration, and exosomes were then observed in the nasal cavity and olfactory nerve 3 h INDD; **(b)** Ex vivo autoradiography of ^64^Cu-labeled exosomes at 3 h post INDD revealed that the highest radioactivity in the olfactory nerve (1 × 10^5^ exosome particles). Radioactivity was also observed in the trigeminal nerves and brain stem, indicating the trigeminal system of INDD. Adapted from Gotoh et al., *Experimental Neurology*; published by Elsevier, 2025

The combination of PET with CT has been effective in precisely localizing a radiotracer’s signal within the bony anatomy of the sinonasal cavity. For instance, Veronesi and colleagues leveraged PET/CT for anatomical segmentation to quantitatively compare the brain concentrations of poly (lactic-co-glycolic acid) (PLGA) nanoparticles (NPs) following intranasal versus intravenous administration. This technique has also provided key insights into delivery strategies [4]. Fukakusa and colleagues used PET/CT with the radiotracer carbon-11-domperidone (¹¹C-domperidone) to show a 2.4-fold increase in N2B targeting when the drug, domperidone, was delivered as a solution with the permeation enhancer lauroylcholine chloride in healthy rats [47]. Similarly, Morin et al. used CT-based anatomical co-registration to validate a novel olfactory drug delivery method that utilizes a cannula to precisely target the olfactory cleft. Their use of the radiotracers fluorodeoxyglucose (^18^F-FDG) and fluorothymidine (^18^F-FLT) demonstrated that this targeted approach achieved superior delivery to the frontal brain regions compared to conventional nasal spray methods [42]. Interestingly, Gotoh et al. showed that intranasally administered ^64^Cu-labeled exosomes were retained in the nasal cavity at 1 h and reached the olfactory nerve by 3 h, with ex vivo autoradiography confirming PET/CT findings and revealing additional signals in the trigeminal nerves and brainstem (Fig. 5) [43].

For imaging brain soft tissue, PET/MRI offers advantages over PET/CT because MRI provides superior anatomical contrast and allows concurrent evaluation of structural integrity, perfusion, and microstructural changes alongside PET-based molecular information. In a key safety assessment, Yuki and colleagues used this multimodality approach to evaluate a nasally administered cationic cholesteryl-group-bearing pullulan (cCHP) nanogel. Their use of MRI confirmed the absence of the PET tracer, ^18^F-FDG, in the OBs and brain, which demonstrated that the nanogel-based vaccine system remained in the nasal passages and did not cross into the CNS [48]. In a contrasting application, Huang et al. leveraged PET/MRI to demonstrate treatment efficacy for acute spinal cord injury in rats using intranasally delivered brain-derived neurotrophic factor-loaded small extracellular vesicles (BDNF-sEVs). The study used an 18-kDa-TSPO PET tracer to quantify the reduction in neuroinflammation, while the MRI documented the consequent morphological repair based on a reduction in the spinal cord lesion cavity [40]. The updated evidence of intranasal imaging with PET is listed in Table 2.

Quantification of N2B transport by PET is subject to partial volume effects leading to spillover from the nasal depot [72]. High radiotracer activity within the nasal cavity immediately after intranasal administration may contribute to the measured activity in adjacent brain regions of interest and potentially overestimate early brain uptake. Co-registration with CT or MRI can improve localization and interpretation, as can careful experimental design which accounts for the effects of radioisotope positron range, scanner resolution, and region of interest geometry and positioning on the magnitude of spillover effects. Spatial resolution and positron energy/range are inversely related, such that higher-energy isotopes like Ga-68 have poorer resolution than lower-energy emitters like F-18 [73]. Spatial resolution is also limited by the scanner’s hardware (e.g. detector material and geometry) and the parameters selected for reconstructing PET images (e.g. spatial resolution, number of iterations) [74]. Finally, the degree of spillover from the nasal cavity to CNS structures is limited by distance, with spillover effects becoming negligible at 3x the scanner’s spatial resolution [72]. Because of this relationship, brain tissues of interest are out of range from significant spillover effects in sufficiently large or otherwise anatomically amenable species, such as the aforementioned study by Smith and colleagues in which the olfactory bulb of nonhuman primates was measured to be more than 4x the full width half maximum (1.4 mm for the Siemens microPET Focus 220) from the nasal cavity tracer depot [45]. Thus, radioisotope selection, scanner resolution, species anatomy, and imaging parameters should all be carefully controlled in INDD experiments utilizing PET to minimize the impact of spillover effects.

## SPECT

Single-photon emission computed tomography (SPECT) is a nuclear imaging technique similar to PET that detects gamma photons emitted during radioactive decay of injected radiotracers rather than positrons. Like PET, SPECT yields functional and physiological information, and when paired with CT, it offers complementary anatomical context. SPECT detects single gamma photons and uses a physical collimator, whereas PET detects coincident 511-keV photon pairs, giving intrinsic electronic collimation along a line of response. SPECT resolution is lower mainly because collimation imposes geometric blur (worse with source–collimator distance) and forces a strong sensitivity–resolution tradeoff, limiting counts and recoverable detail compared with PET. SPECT is performed using a gamma camera comprising a lead collimator which selects photons based on direction and a scintillation detector. The collimator introduces a fundamental trade-off between spatial resolution and sensitivity. During image acquisition, one or more camera heads rotate around the patient to collect multiple projections that are reconstructed into tomographic slices using iterative algorithms [75]. Energy windowing around the photopeak helps suppress Compton scatter, and hybrid SPECT/CT systems provide CT-based attenuation correction and anatomical localization [76]. Advantages of SPECT include relatively low cost and availability of SPECT radionuclides.

Featured SPECT studies are depicted in Table 3. SPECT was leveraged using technetium-99 m (Tc99m) radiotracer to validate the deposition site of a novel INDD device that utilizes laminar flow ejection (LFE) to deliver a bolus of solution to the olfactory cleft. When post-dosing SPECT images were overlayed on anatomical MR images, radiotracer predominantly deposited in the target olfactory cleft region in 7 of 8 human subjects. These results were corroborated via delivery of methylene blue followed by nasal endoscopy to assess regional deposition with greater resolution. Greater than 50% of the administered methylene blue was found in the olfactory cleft for 4 of 5 subjects using the LFE device, whereas a conventional nasal sprayer failed to deliver at least 50% of the methylene blue dose in 5 of 5 of the same participants [49].

**Fig. 6:**
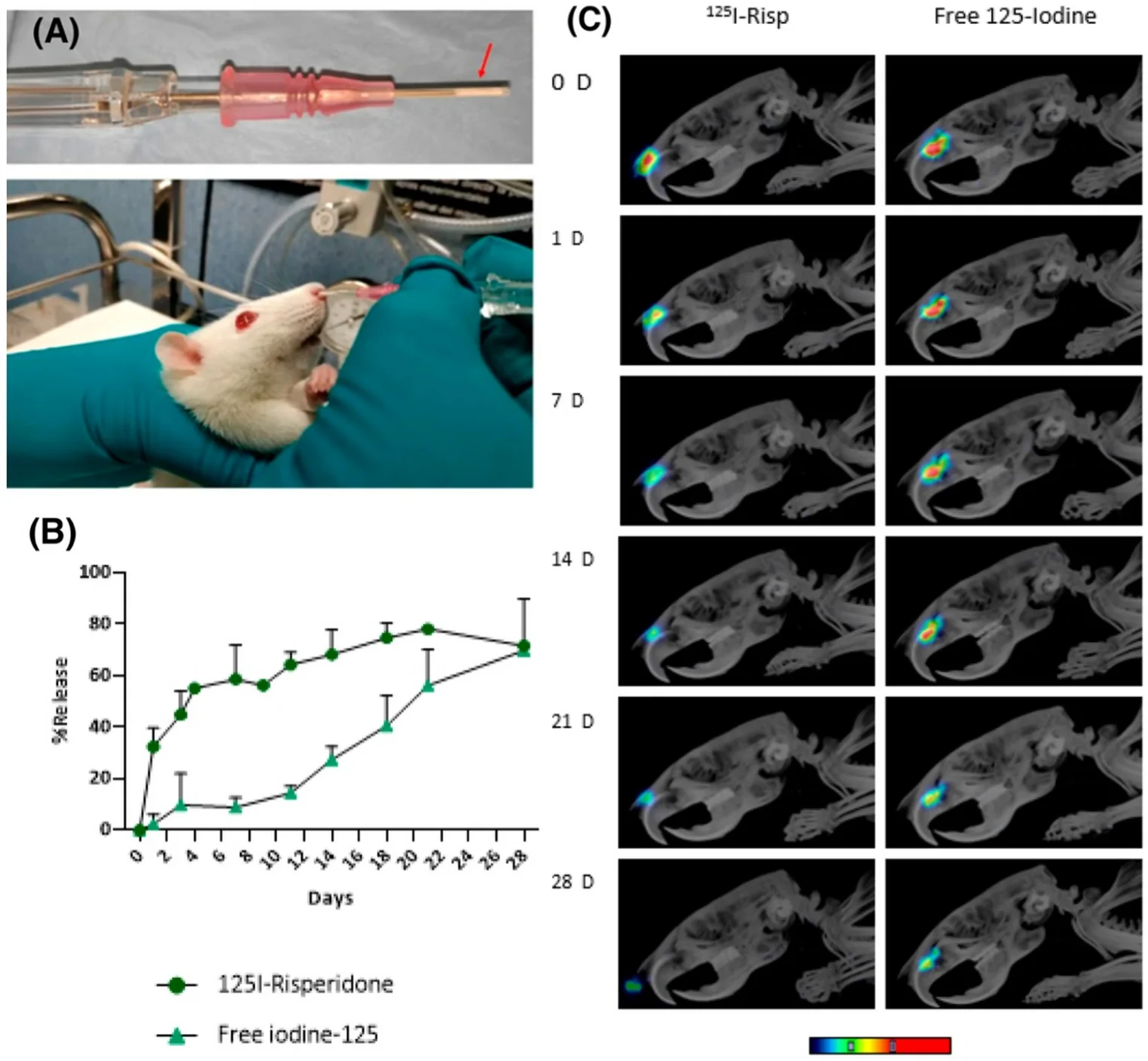
SPECT/CT-based evaluation of implantable nasal rod. **(a)** Placement of a microimplant (red arrow) within a modified intranasal catheter and subsequent administration into the nasal cavity of the rat; **(b)** Quantitative assessment of in vivo radioactivity release from the implant over time, derived from longitudinal SPECT/CT imaging and expressed as mean ± SD; **(c)** Representative SPECT/CT images demonstrating the progressive decline in nasal cavity radioactivity following implantation of a ¹²⁵I-labeled risperidone rod over a 28-day period, with warmer colors indicating higher tracer concentration. Adapted from Simón et al., *Pharmaceutics*; published by MDPI, 2023

In another intranasal delivery study that involved the use of SPECT, the antipsychotic medication risperidone was tracked following release from a rod implanted in the nasal cavity of rats [50]. Risperidone was labeled with I-125 (125-I-RISP) before incorporation into the implant formulation, which allowed for the risperidone release rate to be quantified noninvasively by SPECT over 4 weeks (Fig. 6). Ultimately, 45% of 125-I-RISP was released within 3 days before slowing to a steady release rate, whereas ^125^I (free Iodine 125) implants demonstrated a slow and steady release profile that reached 45% release by 21 days. By combining SPECT with CT, the team was able to validate the placement of the implanted drug delivery system and characterize the release profile over time for differing formulations. Of note, 125-I-RISP was not detected in the brain of implanted rats, argued to be a result of the low concentration of radiotracer released at any one time.

## Fluorescence imaging (FLI)

Fluorescence imaging (FLI) utilizes fluorescence molecules (fluorophores) that absorb light at a particular excitation wavelength and subsequently emit light at a longer wavelength [77]. Dyes, quantum dots, or fluorescent proteins like green fluorescent protein (GFP) are examples of exogenous fluorophores that can be conjugated to drugs, antibodies (or other therapeutics), or expressed in cells to visualize biological structures and processes in vivo. Clinically, FLI is employed in techniques such as fluorescence-guided surgery and endoscopy, where near-infrared dyes such as indocyanine green highlight tumors or blood flow in real time without using ionizing radiation [77]. While FLI provides high spatial resolution for superficial tissues and real-time imaging capability, it suffers from shallow tissue penetration of excitation and emission light (on the order of only a few mm to cm) due to strong photon attenuation (scattering and absorption) by tissues. Additionally, endogenous molecules such as collagen and porphyrins can be excited and fluoresce to cause a substation background autofluorescence, thereby lowering contrast and sensitivity [78]. For these reasons, FLI is rarely encountered in vivo in human studies so all of the studies listed in this section were performed at the preclinical level.

The number of preclinical studies utilizing FLI for the study of INDD has grown rapidly in recent years, a summary of which is provided in Table 4. Both in vivo and ex vivo preclinical approaches have been used and the majority involve nanomedicinal approaches. For instance, Zhao et al. developed a carrier-free nanomedicine (NanoQC) from quercetin self-assembled with polyethylene glycol. When delivered intranasally in mice with 1-methyl-4-phenyl-1,2,3,6-tetrahydropyridine (MPTP)-induced Parkinson’s disease (PD), treated mice outperformed untreated mice in tests of cognitive ability and motor function. In vivo FLI demonstrated maximum signal in the brain at 1 h, and delivery to the substantia nigra and hippocampal regions was corroborated with ex vivo confocal fluorescence microscopy [54].

In another study by Tolomeo et al., human mesenchymal stromal cell-derived extracellular vesicles (MSC-EVs) were tagged with the fluorescent lipophilic dye DiR and delivered intravenously, intratracheally, and intranasally to BALB/c mice. In vivo FLI demonstrated signal retention in the cerebral region of the mice at 3 and 24 h following INDD, whereas ex vivo scanning confirmed high signal in the brain and low signal in the lungs and liver relative to IV and intratracheal (IT) delivery [55]. Brar et al. employed in vivo FLI to study intranasal micelle therapy for treating glioblastoma. Specifically, the authors utilized a block co-polymer methoxy PEG-PLA to encapsulate a conjugate of the tyrosine kinase inhibitor crizotinib with a heptamethine cyanine near-infrared fluorescent dye (mPEG-PLA crizotinib-IR786). Following intranasal delivery of the micelles every day for four days in a Balb/c orthotopic xenograft mouse model of glioblastoma, in vivo FLI on day five demonstrated retention of the micelles in anterior brain regions with reduced accumulation in off-target organs compared to a solution formulation control [51]. In yet another recent example, Bolon et al. used in vivo FLI to compare the uptake of plain polylactic acid NPs versus those synthesized or coated with P188 or P407 poloxamer. The NPs synthesized with P188 poloxamer exhibited the highest in vivo fluorescence intensity immediately following delivery, although this difference was not statistically significant [53].

Recently, Shen et al. combined in vivo FLI with immunofluorescence microscopy (IF) to study the commensal nasal bacterium *Lactobacillus plantarum* WCFS1 (Lp) as a vector for cerebral drug delivery. Following the administration of fluorescein isothiocyanate tracer (FITC)-labeled Lp into the nostrils of C57B/6NTac mice, in vivo FLI and IF collectively demonstrated that the bacteria translocated beyond the OE into the underlying lamina propria and eventually into the OB over 14 days. In contrast, appetite-modulating hormones secreted by the bacteria were observed to localize intracellularly to all three layers (sustentacular cells, OSNs, and basal cells) of the OE, suggesting a different route of nasal uptake relative to the extracellular translocation of the bacterial vector [52]. These results demonstrate how the combination of FLI with ex vivo analytical methods is a useful approach for delineating between intracellular and extracellular uptake pathways.

FLI can also be combined with other imaging modalities to provide improved insight into biodistribution and therapeutic efficacy. For instance, Han et al. studied the INDD of gold nanorods (AuNR _PEG-NH2_) conjugated to a Cy5 fluorescent tracer molecule via FLI, SPECT-CT, and autoradiography. Two weeks after undergoing intracranial GL261 glioma tumor implantation, male and female C57BL/6 mice were inoculated with 20 µL of 300 nM nanorod solution. During ex vivo FLI studies, a higher signal could be seen in frontal brain regions relative to control mice for up to 7 days post-administration. Measurement of coronal brain slices via autoradiography localized uptake to the OBs, which demonstrated up to 8-fold higher uptake relative to other brain regions. Whole body biodistribution studies using SPECT-CT with a [^111^In]diethylenetriaminepentaacetic dianhydride (DTPA)-AuNRP_EG-NH2_ tracer demonstrated nasal localization with off-target accumulation in the stomach and intestines. This biodistribution pattern, along with the presence of the drug in the brain, was further confirmed via gamma counting [56].

**Fig. 7:**
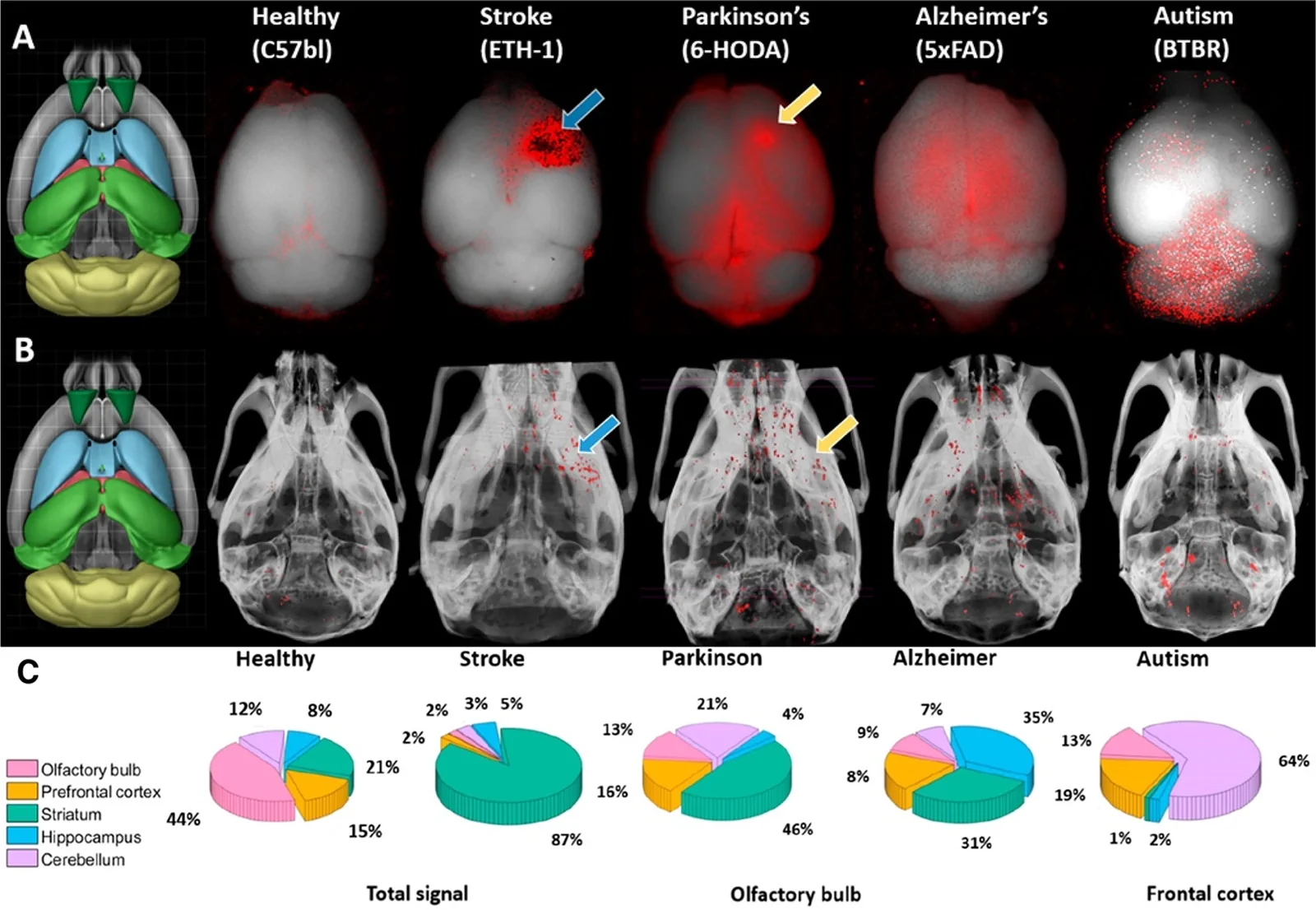
FLI imaging of bone marrow mesenchymal stem cell-derived exosomes (MSC-exos) following intranasal delivery in mouse models of various CNS pathologies. **a)** Ex vivo FLI signal; **b)** In vivo CT signal; **c)** Relative distribution of exosomes among various brain regions for each pathology. Adapted with permission from Perets et al., *Nano Letters*; published by ACS, 2019

In another example, Cheng et al. combined in vivo FLI with MRI to evaluate oxytocin-loaded, macrophage-biomimetic liposomes as a potential AD therapy. Following INDD of the liposomes via pipetting into the nasal cavities of AD mice, apparent brain distribution during in vivo FLI was corroborated by confocal immunofluorescence of hippocampal tissue sections. T2-weighted MRI imaging demonstrated the rebound of hippocampal volumes towards wild-type levels in treated mice with corresponding behavioral improvements in spatial learning and memory tests [57]. Perets et al. used a combination of ex vivo FLI and CT to investigate the INDD of gold nanoparticle-labeled, mesenchymal stem cell-derived exosomes (MSC-exos) for the treatment of stroke, autism, PD, and AD. Ex vivo FLI corroborated in vivo CT imaging evidence that exosomes accumulated in the OBs of healthy mice at 1 h and were gradually cleared over the first 24 h with minimal migration to other brain regions. In contrast, exosomes accumulated over 96 h in pathological CNS tissues of afflicted mice, including the infarcted striatum in the stroke model, the hippocampus in the AD model, the striatum in the PD model, and the cerebellum and prefrontal cortex in the autism model (Fig. 7) [58].

One potential pitfall of FLI is its susceptibility to free-dye artifact, which occurs when the fluorophore detaches from the therapeutic nanocarrier and generates signal apart from where the therapeutic molecule is localized. The likelihood of this confounding complication can be lessened via optimal matching of biologic and nanocarrier characteristics, including the maximization of carrier-fluorophore linkage nondegradability and the minimization of tissue autofluorescence [79]. Delivery of conjugated nanocarrier can be compared to the delivery of free, unconjugated dye as a negative control to confirm that measured signal reflects the intact therapeutic payload, rather than the nonspecific accumulation of free dye. In addition to comparing signal distribution directly while using either in vivo or ex vivo FLI, post-delivery tissue homogenization and subsequent gel electrophoresis can be used to distinguish free dye from intact conjugated dye based upon the expected molecular weight of the labeled molecule [80]. Additionally, thoughtful experimental design using a multipronged approach can provide secondary verification of fluorescence localization. For instance, in the previously discussed study by Cheng and colleagues, the apparent hippocampal distribution of fluorescence agreed with the localization of observed therapeutic effects, including hippocampal volume increases and corresponding memory improvements [57]. Observed fluorescence patterns can also be corroborated using a second imaging modality, such as the comparison performed by Han and colleagues between FLI and SPECT-CT data [56]. Finally, novel nanocarrier technologies can be utilized which require the fluorophore to be intact in order for fluorescent signal to be produced [79].

## Bioluminescence imaging (BLI)

BLI is not used in clinical practice due to the limitations of light scattering and attenuation by the presence of the calvarium, but it is a valuable tool at the preclinical level for furthering the mechanistic understanding of INDD. BLI is an optical technique in which light is produced internally via a chemiluminescent enzyme-substrate reaction (most commonly, firefly luciferase oxidizing luciferin to emit photons) [81]. In practice, cells are genetically engineered to express a luciferase reporter. Upon systemic administration of the luciferin substrate, light is generated in vivo and captured by sensitive cameras. BLI is highly sensitive and virtually background-free, as no external excitation is used, and mammalian tissues have no endogenous luciferase activity [81]. Owing to its high SNR, BLI can detect exceedingly small cell populations (even on the order of single cells in mice) [78, 81]. Another advantage is that bioluminescent light (often in the green-red range) experiences only one-way travel to the detector, thereby yielding deeper effective imaging depth than FLI under comparable conditions. However, the emitted light still undergoes attenuation with tissue depth; so, BLI is mainly useful for small animal and superficial imaging applications [78, 81]. Additionally, BLI lacks clinical translatability – it is essentially restricted to preclinical research because it requires transgenic expression of luciferase or introduction of luciferase-expressing cells.

BLI INDD experiments are summarized in Table 5. Using a combination of BLI, in vivo and ex vivo FLI, and MRI, Villa et al. studied the homing capabilities of gadolinium- and indocyanine green-loaded, canine glioma-derived extracellular vesicles (CG-EVs) in NFkB-luc transgenic mice. First, the authors demonstrated using in vivo and ex vivo FLI that the CG-EVs preferentially accumulated in the brains of healthy mice and in the tumor tissue of mice bearing peripheral subcutaneous gliomas. The authors then intranasally inoculated the transgenic mice with glioma stem cells to generate tumor burden without disrupting the BBB, beyond which tumor growth was tracked using BLI, and gadolinium-loaded CG-EV homing to the tumor site was demonstrated via MRI at five weeks post-intranasal delivery [59].

The lesser sensitivity of BLI detection in vivo compared with ex vivo must be considered when applying BLI to the study of INDD, as demonstrated by Wang et al. in their study of a human neural stem cell (hNSC)-based therapy for an MPTP-induced mouse model of PD. The authors observed that a higher number of hNSCs were needed in rats (1 × 10^6^) compared with mice (1 × 10^5^) for intracranial visualization with BLI at 20 min following intracranial injection. No significant differences in fluorescent intensity could be detected at 2 h post-administration. Following dissection of the brain tissues, fluorescent intensity was found to be significantly elevated compared to controls, suggesting that BLI is limited in its ability to detect signal at deeper distances and lower concentrations in vivo. Subsequent PET/CT imaging following stereotactic injection of ^89^Zr-hNSCs injected into various brain regions of rats could detect 1 × 10^3^ cells in the striatum and 1 × 10^5^ cells in the cortex and hippocampus at 4 h. In contrast, a dose of 2 × 10^6^ cells was required for detection up to 4 h following INDD. Given the resolution limits of PET/CT and BLI methods in vivo, the authors developed a droplet digital PCR (ddPCR) method to amplify and quantify a species-specific gene marker from the stem cell line in postmortem frozen slices, paraffin slices, and whole blood samples. Using immunohistochemistry results as the validation standard, the authors determined that their ddPCR method had a sensitivity of 100–200 cells/4 brain frozen slices [60]. To balance the advantages and disadvantages of various modalities in the study of INDD - such as the trade-off between real-time visualization using BLI, and increased sensitivity of detection using ddPCR - in vivo imaging should be strategically paired with ex vivo techniques at the preclinical research level to continue to improve our understanding of N2B delivery.

## Image-guided drug delivery

Besides the collection and analysis of in vivo imaging data for diagnostic purposes, additional consideration should be given to the ability of imaging principles, including the application of an external magnetic field and pulsed light and sound, to modulate BBB permeability and resulting N2B transport. This concept is called image-guided drug delivery, which broadly refers to the application of imaging technologies to manipulate the delivery of therapeutic payloads in time and space. Image-guided drug delivery goes a step beyond theranostics in which the therapeutic cargo passively doubles as an imaging tracer, to an active process in which imaging is used to direct localization of the therapeutic, manipulate the biological conditions under which it is delivered, or time the release of its payload [82]. Recent studies in INDD which fall under this umbrella are depicted in Table 6, with featured approaches including focused ultrasound, pulsed laser light, and the application of an external magnetic field.

In contrast to the use of standard ultrasound for noninvasive, non-ionizing visualization of anatomy and vascular flow, focused ultrasound (FUS) alters the intensity of the ultrasound waves to produce a therapeutic effect. FUS can directly ablate tissues/lesions using high heat or can facilitate the reversible opening of tight junctions by applying mechanical stress via slight temperature elevation and short-wave pulses. Several variations on FUS have been developed and investigated for a wide variety of CNS conditions, each with distinct advantages and drawbacks. Examples of FUS include the use of high- and low-intensity FUS for stem cell delivery and treatment of essential tremors and PD, respectively; shockwave FUS for the treatment of glioblastoma; MRI-guided FUS for drug delivery to diffuse intrinsic pontine glioma tumors; FUS with gas-filled microbubbles for the treatment of morphine addiction, and for improved INDD of low intensity delivery of dextran across tight junctions [83].

Lin et al. used low-intensity, transcranial planar ultrasound without microbubbles to facilitate tight junction opening in mice following INDD with dextran. Ex vivo FLI of the whole brain and trigeminal nerve demonstrated significantly higher fluorescence in the OB at 4 h in inoculated mice treated with ultrasound compared to inoculated mice that did not receive ultrasound treatment. These results were supported by the dissection, homogenization, and spectrophotometric microplate analysis of various brain regions, which demonstrated significantly higher levels of fluorescence in the OB and trigeminal nerve regions of the brain when ultrasound was used [61]. Thus, supplementation of INDD with FUS is a promising strategy for maximizing N2B delivery when ultrasound parameters are optimized relative to the regional anatomy and drug properties of interest. Building on demonstrations that low-intensity, transcranial planar ultrasound can enhance nose-to-brain transport without exogenous microbubbles, the field can greatly benefit from the development of image-guided, closed-loop protocols that pair distribution imaging with acoustic feedback to individualize sonication and verify regional delivery in real time. In parallel, theranostic extensions using ultrasound-responsive, imageable carriers may enable on-demand release and tracking of small molecules, peptides, NPs, and vectors, positioning FUS as a promising controllable treatment modality for intranasal drug delivery in the future.

Other recent advancements in the study of INDD have focused on the use of pulsed laser light to increase the efficiency of drug delivery. For example, Semyachkina-Glushkovskaya et al. utilized this approach to optimize the delivery of monosialotetrahexosylganglioside (ganglioside GM1)-containing, fluorescently labelled liposomes for the treatment of glioblastoma. The authors first demonstrated that meningeal lymphatic vessels were important for the spreading of liposomes in brain tissues and that following the INDD of liposomes, such spreading is reduced in glioblastoma-afflicted mice compared to healthy controls. Subsequently, the authors used a pulsed laser light treatment immediately following INDD to restore liposome spreading [62]. Notably, the laser treatment itself was an involved procedure requiring a portion of fur and scalp to be removed from the mouse skull before photostimulation. In contrast, the application of transcranial ultrasound in Lin et al. required the shaving of fur but left the scalp itself intact, suggesting that ultrasound is the least invasive of the two approaches for now.

Another approach involves the application of an external magnetic field to increase N2B delivery of magnetoelectric nanoparticles (MENs). Specifically, Pardo et al. conjugated MENs to a near-infrared dye and injected them into the nasal cavities of mice. In vivo FLI demonstrated uptake of the 30 nm MENs into upper brain regions, whereas the addition of a magnetic field resulted in uptake of both 30 and 60 nm MENs into upper brain regions [63].

Similar principles might also be relevant to the development of magnetically guided radiotherapy theranostics. Pandey et al. developed novel pH-responsive, poly-l-lysine-coated Fe_3_O_4_@FePt core shell NPs modified with mitochondria directing triphenylphsphonium ion (TPP) and hypoxic cancer cell directing protein chlorotoxin (CTX) for the treatment of glioblastoma. Ex vivo fluorescence microscopy analysis demonstrated FITC-labeled mito-NPs in the OE and brain tissues following INDD, albeit with significant off-target accumulation in the stomach and intestines [64]. Seino and colleagues likewise developed surface PEGylated, superparamagnetic gold/iron oxide composite NPs and delivered them into the nasal cavities of AD mice. IF demonstrated accumulation of the NPs in senile plaques in the brain [65]. While the in vivo imaging performance of either nanoparticle tracer has yet to be validated, these experiments reflect a larger and growing interest in the development of intranasally delivered, magnetic field-responsive NPs and theranostic probes for use with MRI and magnetic particle imaging.

## Future directions

A deeper understanding of nasal cavity physiology and its intersection with prospective nose-to-brain pitfalls will be important to push the field forward. The complex nasal environment is abundant in opportunities to restrict the amount of nose-to-brain agent available for delivery by trapping, inactivating, or clearing materials before they reach their target site. Materials trapped by the mucosa may be subject to deactivation and/or degradation by drug-metabolizing enzymes, scavenging macrophages, or removal via mucociliary clearance [84]. Unintended absorption by nasal cavity vessels, such as blood vessels or lymphatics that drain to cervical lymph nodes, can further reduce the available drug pool and limit transfer efficiency. The proximity and interwoven nature of these structures are highlighted in Fig. 1. Imaging can be used to mitigate these pitfalls by characterizing the proportion of imaging agent observed at unintended delivery sites and testing approaches to reduce in an iterative manner, particularly nuclear imaging techniques that allow for sensitive quantification. However, lower-resolution imaging techniques may overestimate regional quantification due to residual signal in the nasal cavity and/or signal spillover in these nearby collecting/clearing structures. Thus, efforts to delineate the source of the signal and reduce undesired signals will be valuable to the field. Post-dose nasal rinsing or click-chemistry approaches to imaging agent delivery [85] may help to reduce nonspecific/residual signal and improve signal-to-noise ratios.

Precision medicine will be critical to advancing N2B interventions due to patient anatomical and physiological variability. The impact of nasal anatomy, airflow and mucociliary secretion/clearance dynamics, and various disease states (transient or chronic) on the nasal cycle should be weighed when considering a patient for intranasal treatment. Anatomical imaging (e.g., CT, MRI) can be used to determine whether a patient’s anatomy or physiology will allow for N2B delivery as a treatment strategy, such as screening for septal deviations [86], an obstructive nasal cycle [87], or lesions to delivery conduits such as those observed for the olfactory pathway in AD [88]. A similar approach can be used to tailor strategies to a patient’s unique needs when combined with computational or 3-dimensional models [89, 90]. Finally, functional imaging using labeled tracers (e.g., PET, SPECT) can be an effective means to validate successful N2B delivery and target engagement (e.g. receptor binding) [91], useful for predicting patient responsiveness and selecting from a range of treatment options. Leveraging these tools in combination may accelerate the evaluation of anatomical or disease features that challenge successful intranasal delivery and prospective solutions to address them.

The role of the nasal microbiome as it relates to INDD is an area in need of attention. Significant interpersonal differences exist in this microbiome [92] and were recently found to be associated with neurological health [93, 94]. It would be useful to understand the capacity these populations have for scavenging, metabolizing, or altering the physical-chemical properties and thus behavior of INDD agents through released co-factors or metabolites. Modulating microbial populations in the nasal cavity, or leveraging them for INDD [52], is an area worthy of future exploration.

Personalized interventions and technological advancements will be useful towards addressing the described challenges. Personalized formulations may include constituents that mitigate potential physical/chemical changes to INDD agents in the nasal environment (deactivation, degradation, off-target associations or binding), modulate interactions with entities residing in the nasal cavity (immune cells, microbes, enzymes, tight junctions), or otherwise improve the relatively low delivery efficiency observed with N2B treatments [95]. Nanobody or penetration enhancer incorporation may help to expand the library of effective nose-to-brain agents beyond small molecules and peptides. Similarly, INDD delivery devices and/or techniques that improve deposition at the intended site, reduce dose loss, and are tolerable to the patient should be a focus of development. Advancements in fluid dynamics and aerosolization physics may be useful to this end. With these improvements, repurposing oral or intravenous treatments as nasal formulations will be worthy of exploration (i.e. hallucinogenic substances [96] and neurotransmitter modulators [97] for the treatment of depression and other psychiatric disorders). Imaging can be used to compare delivery strategies and optimize formulations in an iterative manner.

## Conclusions

This review summarizes the literature since the first iteration of Imaging of Intranasal Delivery published in 2020. More work is needed to realize the promise of intranasally delivered therapies for the treatment of CNS diseases, particularly regarding the nasal environment and patient heterogeneity. In vivo imaging can help to better understand these barriers to improving CNS disease therapies.

As the various imaging techniques are refined and applied to INDD, their combined application should balance strengths and weaknesses relevant to the application, including translatability to humans, to accelerate progress towards the development of efficacious and clinically relevant intranasal therapies. Combined PET/MRI offers significant advantages for studying intranasal delivery relative to other modalities, as PET boasts high specificity and signal penetration through attenuating bony structures, while MRI offers high resolution without the risks of ionizing radiation. Further, both modalities are clinically relevant such that work conducted in small animal models, where MR’s resolution remains high at high magnet strengths, can be translated to advancements in humans. The major disadvantage is that hybrid PET/MRI is expensive and not widely available [98]. PET/CT remains a competitive option, as demonstrated by direct evidence of N2B delivery in high-impact articles included in this review. Alternatively, the low translatability of fluorescence and bioluminescence focuses the use of these modalities on answering key questions at the preclinical level.

In addition to the high utility of diagnostic imaging to study INDD in humans and animals, there are also exciting adaptations of these same imaging modalities for therapeutic intervention, including FUS, pulsed laser light, and MR-guided magnetofection. As a future outlook, the concept of theranostics can be applied to INDD, where a positron- or gamma-emitting radiotracer (for PET and SPECT, respectively) is labeled with a beta- or alpha-emitting partner to exert radiation therapy at the target cell/tissue destination. Glioblastomas, meningiomas, or nasal/sinus and other difficult-to-reach cancers may benefit from this application [99, 100].

In conclusion, whereas classic literature overviews play a vital role in establishing the historical timeline and baseline physical properties of intranasal contrast agents, moving the field toward routine clinical translation requires a paradigm shift. The literature from 2020 to 2025 demonstrates that the next generation of nose-to-brain interventions will not merely observe passive transit but could actively engineer delivery via closed-loop image-guided sonication, personalized 3D anatomical casting, and targeted mucosal interface modifications. By framing recent data around these translational variables, this updated overview provides a conceptual framework for clinical trial design and therapeutic optimization for imaging and treatment of complex central nervous system pathologies.

## Data Availability

No datasets were generated or analysed during the current study.

## Abbreviations

AD: Alzheimer’s disease
BBB: Blood brain barrier
BDNF-sEVs: Brain-derived neurotrophic factor-loaded small extracellular vesicles
BLI: Bioluminescence imaging
cCHP: Cationic cholesteryl-group-bearing pullulan nanogel
CEST: Chemical exchange saturation transfer
CG-EVs: Canine glioma-derived extracellular vesicles
CNS: Central nervous system
CSF: Cerebral spinal fluid
CT: Computed tomography
ddPCR: Droplet digital polymerase chain reaction
EMF: External magnetic field
FITC: Fluorescein isothiocyanate tracer
FLI: Fluorescence imaging
fMRI: Functional magnetic resonance imaging
FUS: Focused ultrasound
GM1: Monosialotetrahexosylganglioside
hNSC: Human neural stem cell
INDD: Intranasal drug delivery
IF: Immunofluorescence microscopy
IN: Intranasal
IT: Intratracheal
IV: Intravenous
LCC: Lauroylcholine chloride
LFE: Laminar flow ejection
Lp: *Lactobacillus plantarum* WCFS1
LPNPs: Polymeric micellar nanoparticles
MCA: Middle cerebral artery
MCI: Mild cognitive impairment
MEN: Magnetoelectric nanoparticles
mPEG-PLA: Co-polymer methoxy poly(ethylene glycol)-block-poly(lactic acid)
MPTP: 1-methyl-4-phenyl-1,2,3,6-tetrahydropyridine
MRI: Magnetic resonance imaging
MR: Magnetic resonance
MSCs: Mesenchymal stromal cells
MSC-EVs: Human mesenchymal stromal cell-derived extracellular vesicles
MSC-exos: Mesenchymal stem cell-derived exosomes
N2B: Nose to brain
NPs: Nanoparticles
OB: Olfactory bulb
ODD: Olfactory drug delivery
OE: Olfactory epithelium
OSN: Olfactory sensory neurons
PD: Parkinson’s disease
PET: Positron emission tomography
PLGA: Poly(lactic-co-glycolic acid)
SNR: Signal-to-noise ratio
SPECT: Single-photon emission computed tomography
Tc99m: Technetium-99m
TSPO: Translocator protein
WMHV: White matter hyperintensity volume
[^18^F]FB-insulin: Fluorine-18-labeled insulin
^18^F-FDG: Fluorodeoxyglucose
^18^F-FLT: Fluorothymidine
^18^F-PBR06-PET: 18F-Fluoroacetyl-N-(2,5-dimethoxybenzyl)-2-phenoxyaniline
[^68^Ga]Ga-NOTA-insulin: Gallium-68-labeled insulin
125-I-RISP: Iodine 125-labeled risperidone
^125^I: Free iodine 125
